# Bridging the Gap: The PrEP Cascade Paradigm Shift for Long-Acting Injectable HIV Prevention

**DOI:** 10.3390/v18030336

**Published:** 2026-03-09

**Authors:** Adrian Charles (AC) Demidont

**Affiliations:** Nyx Dynamics, LLC, Fairfield, CT 06824, USA; acdemidont@nyxdynamics.org; Tel.: +1-203-247-1177

**Keywords:** HIV prevention, pre-exposure prophylaxis, long-acting injectable, cabotegravir, lenacapavir, implementation science, care cascade, health equity, clinical decision support

## Abstract

Long-acting injectable HIV pre-exposure prophylaxis (LAI-PrEP) demonstrates superior efficacy and persistence compared to daily oral PrEP. However, real-world implementation reveals that only 52.9% of prescribed individuals initiate treatment with their first injection. This implementation barrier stems from a fundamental mismatch between the traditional PrEP cascade—designed for oral formulations allowing same-day initiation—and LAI-PrEP’s unique requirements involving a 2–8 week “bridge period” between prescription and first injection to establish HIV-negative status. We synthesize data from major clinical trials (HPTN 083, HPTN 084, PURPOSE-1/2; >15,000 participants) with real-world implementation studies to characterize bridge period navigation as the critical implementation barrier. This review proposes a reconceptualized PrEP cascade explicitly recognizing the bridge period as a distinct, measurable step requiring dedicated management strategies. We examine pharmacological bases for conservative initiation protocols, quantify population-specific barriers to bridge period completion, and synthesize evidence on strategies to improve initiation success. This paradigm shift from individual behavioral adherence to structural factors within the healthcare system requires parallel innovations in cascade conceptualization, measurement frameworks, and implementation approaches. Addressing this structural barrier is essential to translate LAI-PrEP’s extraordinary clinical efficacy (>96%) into meaningful public health impact, particularly for populations experiencing the highest HIV burden.

## 1. Introduction

### 1.1. Evolution of HIV Prevention

Over three decades of antiretroviral research have fundamentally transformed HIV prevention, evolving from early monotherapy approaches to combination regimens and now to long-acting injectable formulations. This evolution reflects a core principle: HIV prevention effectiveness depends not only on pharmacological efficacy but on how well prevention modalities fit into people’s lives. Daily oral pre-exposure prophylaxis (PrEP) with tenofovir/emtricitabine demonstrates 44–92% efficacy depending on adherence levels and populations studied [1,2]. However, oral PrEP’s effectiveness in real-world settings is limited by the daily decision-making required for adherence. Long-acting injectable PrEP addresses this by shifting adherence from individual daily behavioral choices to structured healthcare systems.

### 1.2. Clinical Efficacy: Robust Evidence Across Diverse Populations

Clinical trials have consistently demonstrated remarkable efficacy for LAI-PrEP across diverse populations. HPTN 083 enrolled 4566 cisgender men who have sex with men (MSM) and transgender women, demonstrating 66% superior efficacy of cabotegravir compared to oral TDF/FTC [3]. HPTN 084 enrolled 3224 cisgender women in sub-Saharan Africa and demonstrated 89% superior efficacy, with early trial termination for efficacy [4].

The PURPOSE program represents the most comprehensive HIV prevention trial program conducted to date. PURPOSE-1 enrolled 5338 cisgender women in South Africa and Uganda, with zero HIV infections in the lenacapavir arm—representing >96% efficacy versus background incidence [5]. PURPOSE-2 enrolled 3265 participants across gender identities (cisgender men, transgender women, transgender men, and gender-diverse persons), demonstrating consistent 96% efficacy across all groups [6]. PURPOSE-3, PURPOSE-4, and PURPOSE-5 are evaluating historically underrepresented populations, including U.S. women of color, people who inject drugs, and diverse global key populations [7].

Phase 1 studies of once-yearly lenacapavir demonstrated plasma concentrations above efficacy thresholds for ≥56 weeks, with Phase 3 evaluation underway [8]. These data establish clinical efficacy across diverse populations. Supplementary File S1 provides detailed clinical trial evidence and safety considerations.

### 1.3. Safety Considerations: Lessons from Islatravir

The development and discontinuation of islatravir for PrEP demonstrates that long-acting formulations require heightened safety monitoring given their pharmacokinetic irreversibility [9,10]. Conservative initiation protocols for cabotegravir and lenacapavir reflect this principle: the bridge period between prescription and injection is part of a safety framework for medications that cannot be rapidly removed from the body. Supplementary File S1 details safety considerations relevant to bridge period management.

### 1.4. Implementation Reality: The Critical Gap

Despite extraordinary clinical efficacy, real-world LAI-PrEP implementation faces a significant paradox. In the CAN Community Health Network study—one of the largest real-world LAI-PrEP implementation cohorts—only 52.9% of individuals prescribed LAI-PrEP received their first injection [11]. This 47% attrition occurs before the first dose is administered, among individuals who have already navigated awareness, eligibility assessment, and clinical decision-making.

In contrast, persistence data from Trio Health demonstrate that 81–83% of individuals receiving at least one cabotegravir injection remain engaged with continuing care [12]. This striking contrast reveals a fundamental structural mismatch: LAI-PrEP addresses oral PrEP’s adherence challenge but creates a new structural barrier during bridge period navigation.

### 1.5. Scope and Objectives

This review proposes that bridge period navigation represents the critical implementation barrier for LAI-PrEP, distinct from well-characterized barriers to oral PrEP persistence. We synthesize clinical trial evidence with implementation data to (1) characterize the bridge period as a distinct implementation stage, (2) quantify population-specific barriers, (3) synthesize evidence on strategies to improve bridge period completion, and (4) identify research priorities for translating LAI-PrEP’s clinical efficacy into public health impact.

Prior reviews of LAI-PrEP have predominantly focused on clinical trial efficacy, pharmacokinetic profiles, and user acceptability, providing essential evidence of LAI-PrEP’s clinical promise. Implementation-focused reviews have addressed oral PrEP cascade barriers, including awareness gaps, willingness, and post-initiation persistence challenges. However, existing reviews have not identified or characterized the bridge period as a distinct cascade step, nor have they proposed standardized monitoring metrics for pre-initiation attrition. This review contributes three novel elements that extend beyond prior literature: (a) formal recognition and definition of the bridge period as a measurable cascade step, creating a framework for systematic measurement and intervention; (b) standardized bridge period monitoring metrics enabling cross-site comparison and quality improvement; and (c) population-stratified bridge period attrition projections derived from convergent evidence and validated computationally in the companion paper [13], providing the first quantitative estimates of population-specific implementation gaps for LAI-PrEP.

### 1.6. Convergent Evidence on Primary Barriers

Across diverse implementation settings, a consistent pattern has emerged: LAI-PrEP’s primary implementation barrier occurs before the first injection, during bridge period navigation. This fundamentally differs from oral PrEP, where the primary barrier occurs after initiation during persistence. This convergent evidence across multiple implementation contexts supports reconceptualizing the PrEP cascade to explicitly recognize bridge period navigation as a distinct implementation step.

### 1.7. Analytical Approach

This review employs a structured evidence synthesis methodology, drawing on implementation science frameworks to characterize bridge period navigation as a distinct implementation barrier for LAI-PrEP. While not a formal systematic review, we describe our search strategy, inclusion criteria, evidence synthesis approach, and conceptual framework to ensure analytical transparency.

#### 1.7.1. Search Strategy

We searched PubMed, Embase, and ClinicalTrials.gov from January 2012 (corresponding to the first PrEP approval) through December 2025, using combinations of the following terms: “long-acting injectable”, “pre-exposure prophylaxis” or “PrEP”, “cabotegravir”, “lenacapavir”, “implementation”, “care cascade”, “bridge period”, “injection initiation”, and “HIV prevention”. We supplemented database searches with forward and backward citation tracking from landmark trials (HPTN 083, HPTN 084, PURPOSE-1, PURPOSE-2) and real-world implementation studies. We also reviewed conference abstracts from CROI, IAS, and IDWeek (2022–2025) and accessed publicly available regulatory documents, WHO guidelines, and CDC recommendations.

#### 1.7.2. Inclusion Criteria

Evidence was included if it met one or more of the following criteria: (1) reported clinical efficacy or safety data for injectable PrEP formulations (cabotegravir or lenacapavir); (2) described real-world implementation outcomes for LAI-PrEP, including initiation rates, persistence, or barriers; (3) characterized population-specific factors affecting PrEP cascade progression; (4) evaluated interventions to improve PrEP initiation, navigation, or retention; or (5) provided foundational data on the HIV prevention cascade framework. We excluded studies focused exclusively on oral PrEP adherence without relevance to injectable formulation implementation, studies limited to pharmacokinetic modeling without clinical or implementation endpoints, and commentaries or editorials lacking original data or novel conceptual contributions.

#### 1.7.3. Evidence Synthesis Approach

We employed a convergent triangulation approach, synthesizing findings from three independent evidence streams: (1) randomized clinical trial data establishing LAI-PrEP efficacy across populations (>15,000 participants across HPTN 083/084 and PURPOSE-1/2); (2) real-world implementation cohort data quantifying bridge period attrition (primarily CAN Community Health and Trio Health datasets); and (3) population-specific implementation studies documenting barriers and facilitators across diverse settings and key populations. Evidence from each stream was classified into tiers reflecting analytical rigor. Tier 1 evidence comprised randomized controlled trials and large prospective cohorts with pre-specified endpoints (e.g., HPTN 083/084 efficacy data, CAN Community Health initiation rates). Tier 2 evidence comprised observational studies, cross-sectional surveys, and implementation evaluations with defined methodologies (e.g., Trio Health persistence data, population-specific barrier assessments). Tier 3 evidence comprised conference abstracts, policy documents, expert consensus, and emerging data from ongoing trials. Our companion computational validation study details this evidence tier classification and its application to clinical decision support algorithm development [13].

For each key finding, we sought corroboration across at least two independent evidence streams before characterizing findings as convergent. Where evidence was limited to a single stream or where findings conflicted across streams, we noted these limitations explicitly. Population-specific projections of bridge period attrition were derived by applying documented barrier amplifiers to the baseline attrition rate of 47% observed in the CAN Community Health cohort. These derivations are presented transparently in Section 3 and validated computationally in the companion paper [13].

#### 1.7.4. Conceptual Framework

This review is organized around a reconceptualized PrEP cascade framework that explicitly recognizes bridge period navigation as a distinct implementation step between prescription and injection initiation. This framework draws on Proctor et al.’s implementation outcomes taxonomy, distinguishing between clinical effectiveness (demonstrated by trials) and implementation effectiveness (determined by real-world delivery systems). The bridge period concept operationalizes the gap between these two domains: LAI-PrEP’s clinical efficacy is established, but implementation effectiveness depends on successfully navigating structural barriers during the bridge period. By identifying bridge period navigation as the rate-limiting step in the LAI-PrEP cascade, this framework redirects attention from individual behavioral factors (the dominant paradigm for oral PrEP) to structural and system-level factors amenable to healthcare delivery intervention.

## 2. The Reconceptualized PrEP Cascade: Making the Bridge Period Visible

### 2.1. Traditional vs. LAI-PrEP Care Cascades

**Figure 1:**
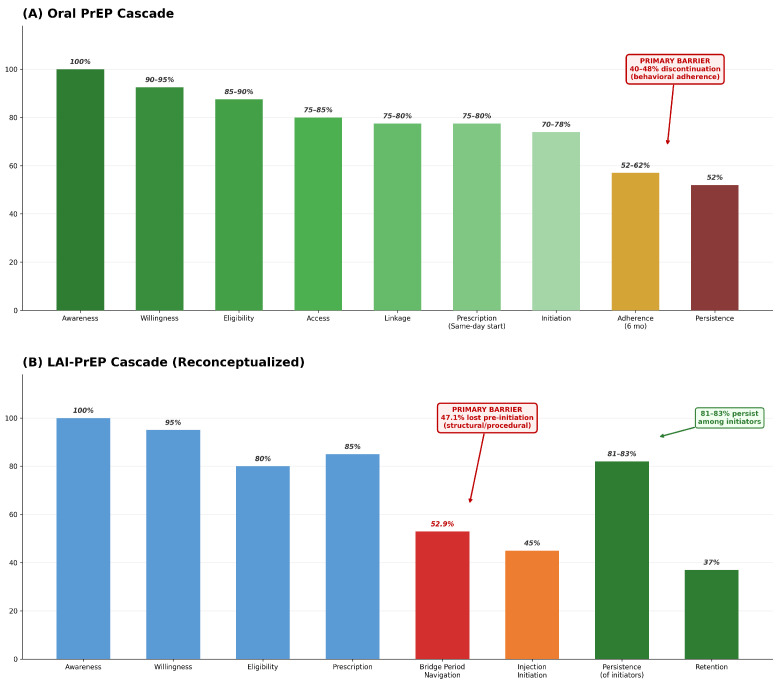
Oral PrEP and LAI-PrEP Cascade Comparison. (**A**) The oral PrEP cascade enables same-day initiation with sequential progression through nine steps. The primary implementation barrier occurs post-initiation, with 40–48% discontinuation due to daily pill burden and adherence challenges. Population-specific discontinuation varies: transgender individuals (70%) [16], young MSM (44–69%), and cisgender women (48–56%). Data sources: HPTN 083 (n = 4566) [3], HPTN 084 (n = 3224) [4], and PrEP cascade framework [14]. (**B**) The reconceptualized LAI-PrEP cascade introduces a bridge period between prescription and first injection as a distinct implementation step. Bridge period navigation represents the critical barrier, with 47.1% of prescribed individuals not receiving their first injection due to HIV testing delays, insurance authorization, and appointment coordination challenges. This represents a fundamental shift from oral PrEP’s post-initiation behavioral barrier to a pre-initiation structural barrier. Those successfully navigating the bridge show superior persistence (81–83%) compared to oral PrEP (52%). Data sources: CAN Community Health Network (47.1% attrition) [11], Trio Health (81–83% persistence) [12], and HPTN 083/084 and PURPOSE-1/2 (efficacy) [3,4,5,6].

The traditional HIV PrEP cascade, developed for oral formulations, consists of sequential steps: awareness, willingness, prescription, initiation, and persistence [14]. In this model, prescription and initiation occur simultaneously or within days; individuals receive a prescription and begin daily oral PrEP immediately, with protective drug levels achieved within 7 days for receptive anal exposure or 21 days for receptive vaginal exposure [15] (Figure 1).

LAI-PrEP introduces a fundamentally different implementation structure. A substantial temporal and procedural gap exists between prescription and first injection. Unlike oral PrEP’s same-day start capability, LAI-PrEP requires confirmation of HIV-negative status through testing with appropriate window period considerations, creating a “bridge period” between clinical prescription decision and treatment initiation.

### 2.2. The Bridge Period: Definition and Components

The bridge period encompasses all activities and time intervals between the clinical decision to prescribe LAI-PrEP and the first injection administration. Bridge period activities include:

**Core requirements:**

- Baseline HIV testing (antigen/antibody test within 7 days of planned injection);
- Additional HIV-1 RNA testing if recent exposure or transitioning from oral PrEP;
- Injection appointment coordination and attendance;
- Insurance authorization (when required).

**Extended bridge period factors:**

- Repeat HIV testing if initial testing predates injection appointment by >7 days;
- Optional oral lead-in period (for cabotegravir tolerability assessment);
- Insurance authorization appeals or delays;
- Individual scheduling barriers;
- Transportation or logistical obstacles.

The bridge period creates a unique structural vulnerability: individuals remain motivated for prevention (having navigated awareness, willingness, and clinical eligibility) but lack protection while navigating procedural requirements. HIV acquisition during bridge period represents a system failure specific to LAI-PrEP that does not occur with oral formulations.

The structural contrast between oral and LAI-PrEP cascades is fundamental: oral PrEP’s primary barrier occurs post-initiation (adherence), while LAI-PrEP’s primary barrier occurs pre-initiation (bridge period). This distinction has direct implications for intervention design and resource allocation (Table 1).

### 2.3. Proposed Reconceptualized Cascade

We propose an LAI-PrEP cascade explicitly recognizing bridge period navigation as a distinct implementation step:

1. **Awareness**: Knowledge that LAI-PrEP exists as a prevention option;
2. **Willingness**: Interest in injectable formulations as a viable approach;
3. **Eligibility**: Clinical criteria and baseline testing requirements met;
4. **Prescription**: Clinical decision to initiate LAI-PrEP;
5. **Bridge Period Navigation**: Successful completion of requirements between prescription and injection:
  - HIV testing completion and negative result within the appropriate window;
  - Injection appointment scheduling and attendance;
  - Financial/insurance barrier resolution;
  - Optional oral lead-in period completion;
6. **Injection Initiation**: Receipt of first LAI-PrEP injection;
7. **Persistence**: Continued receipt of subsequent injections per protocol.

**Figure 2:**
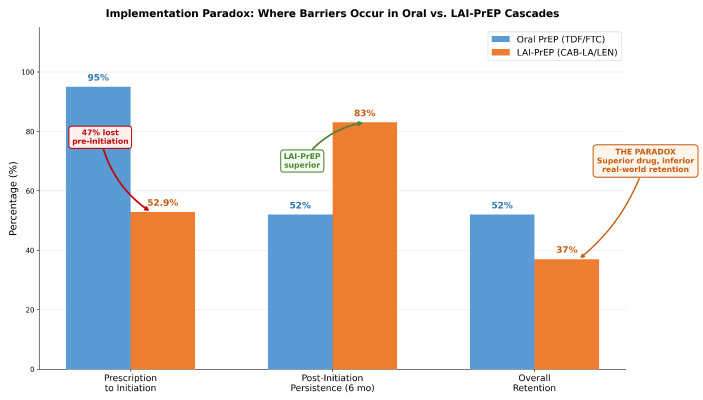
Implementation paradox: where barriers occur. Left: Oral PrEP cascade shows wide entry (same-day initiation), with 40–48% discontinuation post-initiation. Right: LAI-PrEP cascade shows a narrow entry (2–8 week bridge period), eliminating 47% pre-initiation, but 81–83% persistence among those initiating. LAI-PrEP solves oral PrEP’s behavioral adherence problem but introduces a structural access barrier. This fundamental difference requires distinct implementation strategies focused on system-level rather than individual-level interventions. Data sources: HPTN 083/084, CAN Community Health Network, Trio Health.

This reconceptualized cascade makes visible where 47% of LAI-PrEP candidates currently experience attrition. Naming bridge period navigation as a distinct cascade step creates accountability for measurement and intervention at this clinical juncture (Figure 2).

### 2.4. Measurement Implications

The reconceptualized cascade requires new measurement approaches:

**Bridge period success rate**: Percentage of prescribed individuals receiving first injection (current baseline: 53%; potential target: ≥75%).

**Bridge period duration**: Median time from prescription to injection (current variable; potential target: <14 days).

**Attrition causes**: Categorization of why individuals do not complete the bridge period (testing delays, insurance barriers, appointment no-shows, individual decision, loss to follow-up).

**Population-stratified metrics**: Bridge period completion rates for key populations (MSM, women, people who inject drugs, adolescents, transgender individuals).

### 2.5. Implementation Monitoring Framework

Programs operationalizing the reconceptualized cascade may track core metrics:

1. **Bridge period success rate**: Prescribed to injection ratio (baseline: 53%; target: ≥75%);
2. **Time to injection**: Median days from prescription to first injection (target: <14 days for ≥75% of individuals);
3. **Attrition characterization**: Categorized causes of bridge period incompletion;
4. **Population-stratified completion**: Success rates by key populations;
5. **Oral-to-injectable transition rate**: Proportion initiated via direct transition from oral PrEP;
6. **Enhanced testing utilization**: Percentage receiving HIV-1 RNA testing at baseline;
7. **Navigation program reach**: Proportion of prescriptions referred to support services.

These metrics enable programs to identify bottlenecks, compare performance across sites, evaluate intervention effectiveness, and monitor whether LAI-PrEP implementation reduces or exacerbates HIV prevention disparities.

## 3. Population-Specific Bridge Period Barriers

Population-specific bridge period attrition projections are derived from a transparent analytical chain. The baseline attrition rate of 47% is established from the CAN Community Health cohort [11], which represents the largest real-world LAI-PrEP implementation dataset. For each population, we apply documented barrier amplifiers—structural factors with evidence of increased attrition beyond the general population baseline—to project population-specific completion rates. These amplifiers are drawn from Tier 1 and Tier 2 evidence sources as classified in Supplementary File S2 Intervention Inventory.

For adolescents, the 47% baseline attrition is amplified by documented developmental factors (temporal discounting reducing follow-through on delayed outcomes), privacy barriers (insurance explanation of benefits concerns documented by [17,18] and limited autonomous healthcare navigation, yielding a projected completion rate of 30–40% (i.e., 60–70% attrition). For women, intersecting structural barriers including transportation, childcare, and medical mistrust (39% citing side effect concerns as primary barrier) amplify baseline attrition to yield projected 40–50% completion. For PWID, the most severe amplification occurs through criminalization-related care avoidance (17% avoiding care entirely), housing instability, and extremely low baseline awareness (40% aware of PrEP, 2% using it), projecting 20–30% completion. Each projection is cross-referenced and validated computationally in the companion paper [13], which tests these estimates across scales up to 21.2 million simulated patients. Table 2 summarizes the key evidence supporting population-specific barrier characterization and projected bridge period outcomes.

### 3.1. Adolescents (Ages 16–24)

Adolescents face unique developmental and structural barriers to bridge period completion despite demonstrating comparable pharmacokinetics and efficacy to adults. Developmental factors, including temporal discounting, create challenges for navigating multi-week delays between prescription and injection. Privacy concerns are substantial: surveys of Black female adolescents documented that concerns about parental discovery through insurance explanation of benefits created reluctance to initiate despite high HIV risk [17]. The bridge period amplifies these concerns through multiple visits and appointments. Based on general population attrition (47%) and adolescent-specific barriers, bridge period completion in adolescents is projected at 30–40%. Companion computational validation at UNAIDS target scale (21.2 million individuals) confirmed these estimates and demonstrated that evidence-based interventions yield 147% relative improvement for adolescents, the second-largest gain among all populations modeled [13]. Detailed adolescent-specific considerations are presented in Supplementary File S2.

### 3.2. Women

Women, particularly Black and Latina women, navigate intersecting structural barriers, including transportation challenges, childcare responsibilities, and competing caregiving demands that complicate bridge period completion [17]. Medical mistrust rooted in historical and ongoing experiences of medical racism manifests as concerns about side effects (identified by 39% as the primary barrier) and skepticism toward prevention recommendations. Clinical trials demonstrated 89% efficacy among cisgender women (HPTN 084) [4] and zero infections among 5338 women (PURPOSE-1) [5], but these results occurred in research settings with intensive support. PURPOSE-3 will provide real-world implementation data for U.S. women of color. Computational validation projects 40–50% bridge period completion for women, with targeted interventions addressing transportation, childcare, and medical mistrust barriers, producing meaningful improvement [13]. Detailed considerations are in Supplementary File S2.

### 3.3. People Who Inject Drugs (PWIDs)

PWIDs encounter perhaps the most severe structural barriers to bridge period navigation. Criminalization of drug use creates fear of legal consequences and healthcare discrimination, with 17% of PWID avoiding care entirely [38]. Housing instability affects substantial proportions, creating cascading barriers, including a lack of stable contact information for appointment reminders and transportation challenges. PrEP awareness among PWID remains low (40% in Los Angeles/San Francisco surveys), with only 2% currently using it despite indicated need [18]. Syringe service programs represent the most promising delivery setting but face resource constraints limiting bridge-period support [55]. Multiple intersecting barriers project bridge-period completion in PWID at 20–30%. Companion computational validation demonstrated that PWID, despite having the lowest baseline success rate (10.36%), showed the greatest relative improvement (265%) with evidence-based interventions—indicating that targeted bridge period support can narrow rather than widen health equity gaps [13]. PURPOSE-4 will provide critical implementation evidence. Detailed PWID implementation considerations are in Supplementary File S2.

### 3.4. Other Key Populations

Transgender women demonstrated excellent efficacy results (HPTN 083, PURPOSE-2) [3,6] but face healthcare discrimination and economic marginalization, complicating bridge period navigation. MSM show the highest current LAI-PrEP uptake yet experience 47% bridge-period attrition [11], indicating a substantial improvement opportunity. PURPOSE-1 included pregnant and lactating individuals from study inception [5], introducing bridge period considerations, including pregnancy testing requirements and competing prenatal care priorities. Detailed population-specific information is in Supplementary File S2.

### 3.5. Equity Implications

Bridge period barriers create health equity risks. As structural barriers affect all populations but resources to address them are unequally distributed, LAI-PrEP implementation risks widening rather than narrowing HIV prevention disparities. Populations experiencing the highest HIV incidence and the lowest healthcare access encounter the greatest bridge period barriers. Yet these same populations benefit most from LAI-PrEP’s independence from daily adherence. Prioritizing bridge period support for populations experiencing HIV-related disparities is essential to ensure LAI-PrEP implementation reduces inequities rather than exacerbating them.

### 3.6. Global Implementation Context

Global LAI-PrEP implementation introduces additional complexities beyond U.S. settings, including medication cold chain requirements, healthcare workforce capacity, and regional policy considerations. Sub-Saharan Africa accounts for 62% of the global PrEP need but faces heightened structural barriers. Detailed global implementation considerations are in Supplementary File S3.

### 3.7. Affordability and Access as Antecedent Barriers

The bridge-period strategies described in this review assume that LAI-PrEP has been made accessible to the patient. Medication cost represents a fundamental antecedent barrier that determines whether bridge period management becomes relevant. Lenacapavir carries a US list price of approximately $28,218 annually ($14,109 per semi-annual injection) [56], compared to generic oral tenofovir/emtricitabine at approximately $30–60 per month. Long-acting cabotegravir, although approved, remains largely unavailable for PrEP across most European settings due to cost–benefit determinations relative to generic oral alternatives. HIV prevention achieves population-level public health impact only when extensively adopted, making affordability a prerequisite for the implementation strategies proposed herein.

Voluntary licensing agreements through the Medicines Patent Pool may reduce LAI-PrEP costs in 90 low- and middle-income countries, and patient assistance programs enable access in some high-income settings. However, until pricing barriers are resolved through policy-level intervention—distinct from the clinical and system-level interventions addressing bridge period navigation—LAI-PrEP’s superior efficacy cannot translate into proportional public health impact. The bridge period framework and strategies presented in this review become actionable in settings where the access barrier has been addressed, and will be increasingly relevant as cost barriers are reduced through generic manufacturing, voluntary licensing, and national reimbursement decisions.

## 4. Evidence-Based Strategies to Improve Bridge Period Completion

Evidence-based strategies to improve bridge period completion operate across three complementary mechanisms—elimination, compression, and navigation—with effect sizes drawn from our evidence tier classification. Tier 1 evidence (RCTs and large prospective cohorts) supports oral-to-injectable transitions as the highest-impact strategy, with initiation rates of 85–90% compared to 53% for PrEP-naive individuals. Tier 2 evidence (observational studies and implementation evaluations) documents accelerated testing pathways reducing bridge period duration from 45 days to 10–14 days, and patient navigation programs achieving 1.5-fold improvement in PrEP initiation. Tier 3 evidence (conference abstracts and emerging data) informs system-level integration approaches, including pharmacist-led delivery and harm-reduction co-location models. Table 3 presents the complete evidence tier classification for all 21 interventions, with documented evidence sources and estimated effect sizes. The companion computational validation paper [13] details how these interventions are operationalized through a clinical decision support algorithm with mechanism diversity scoring.

### 4.1. Eliminating the Bridge Period: Oral-to-Injectable Transitions

The most effective bridge period management strategy is complete elimination through seamless transitions from oral PrEP to LAI-PrEP. Individuals engaged in oral PrEP demonstrate prior prevention motivation, established provider relationships, recent negative HIV testing, and proven ability to navigate healthcare systems. Transitioning directly from oral to injectable PrEP eliminates multiple bridge period barriers, including extended HIV testing requirements and the need for new provider engagement.

Real-world data from HIV treatment populations demonstrate that oral-to-injectable transitions achieve substantially higher injection initiation rates (approximately 85–90%) compared to PrEP-naive individuals (53%) [11,12], as individuals already engaged in oral PrEP have overcome multiple implementation barriers. Implementation strategies include same-day transition protocols for individuals with current HIV testing, proactive discussions about switching to normalize injectable options, streamlined insurance authorization for switches, and provider education on simplified transition protocols.

Approximately 50% of oral PrEP users discontinue within 6–12 months, creating a secondary target population potentially highly motivated to switch modalities addressing adherence challenges. Re-engagement of former oral PrEP users with LAI-PrEP represents an important implementation opportunity. Computational validation classified oral-to-injectable transitions as a Tier 1 strategy with the highest projected impact on bridge period success rates across all populations modeled [13].

### 4.2. Compressing the Bridge Period: Accelerated Diagnostic Pathways

When oral-to-injectable transitions are not possible, accelerated HIV testing strategies can shorten the bridge period duration. Current CDC guidelines recommend antigen/antibody testing alone for current oral PrEP users, enabling same-visit transition. For PrEP-naive individuals, dual testing (antigen/antibody + RNA) detects acute infections earlier, reducing the window period from 45 days to 10–14 days and enabling bridge completion within 2–3 weeks.

Point-of-care RNA platforms providing results within 90 min enable single-visit dual testing where available. Laboratory-expedited processing of baseline tests can reduce result turnaround to 24–48 h. Risk-stratified testing approaches reserve comprehensive RNA testing for the highest-risk scenarios while maintaining timely initiation for lower-risk individuals.

WHO’s July 2025 guidance recommends simplified HIV testing for LAI-PrEP initiation, reflecting a public health approach that prioritizes access over maximal risk reduction when resource constraints exist [57]. This approach reflects different risk-benefit calculations in resource-limited versus well-resourced settings.

### 4.3. Navigating the Bridge Period: Patient Navigation Programs

When complete elimination or compression is not feasible, intensive navigation support improves bridge period completion. Patient navigation uses trained personnel to identify and mitigate financial, cultural, logistical, and educational barriers to healthcare access. San Francisco navigation programs demonstrated a 1.5-fold increase in PrEP initiation rates, while cancer care navigation meta-analyses show 10–40% improvement in treatment initiation, with the greatest benefits for disadvantaged populations [59,62,68].

Bridge period navigation approaches include dedicated navigators supporting prescription-to-injection transition, text message-based appointment reminders and support, peer navigation leveraging lived experience, and population-tailored approaches addressing specific barriers for adolescents, women, PWID, and transgender individuals.

Financial barriers during the bridge period extend beyond medication costs to transportation, childcare, and opportunity costs. System-level interventions, including telemedicine integration for counseling, pharmacist-led LAI-PrEP delivery in community settings, harm reduction service integration for PWID, and mobile clinic models, can reduce bridge period completion barriers (Table 3). The companion computational validation operationalizes these strategies through a clinical decision support algorithm incorporating all 21 evidence-based interventions with mechanism diversity scoring to prevent redundant recommendations [13]. Detailed methodological notes on intervention effect calculation, mechanism classification, and evidence gaps are in Supplementary File S2.

## 5. Research Priorities

Translating LAI-PrEP’s clinical efficacy into public health impact requires systematic research across multiple domains. Priority areas include: (1) real-world bridge period measurement in diverse settings including resource-limited contexts, (2) population-specific implementation studies emerging from PURPOSE-3/4/5 and HPTN 102/103, (3) comparative effectiveness of testing acceleration and navigation strategies, (4) economic evaluation of bridge period interventions across healthcare system types, and (5) explicit equity monitoring systems ensuring LAI-PrEP implementation reduces rather than exacerbates HIV prevention disparities. Ongoing implementation research, including the EquiPrEP protocol for an equity-focused LAI-PrEP rollout [76], will generate critical evidence. Notably, successful models from hepatitis C direct-acting antiviral implementation among PWID—where simplified treatment achieved >95% cure rates in active injection drug users [77,78]—provide an instructive precedent for LAI-PrEP implementation in this population. A comprehensive research agenda is in Supplementary File S5.

## 6. Conclusions

Long-acting injectable pre-exposure prophylaxis represents a transformative advance in HIV prevention, demonstrating >96% efficacy across diverse populations [5,6], superior persistence compared to oral PrEP (81–83% vs. ∼52%) [12], and strong user preference (67% favor injections over daily pills). However, this clinical success cannot translate into a meaningful public health impact if nearly half of the individuals prescribed never receive their first injection.

This review fundamentally reframes the LAI-PrEP implementation challenge. LAI-PrEP’s structural advantage shifts the implementation question from individual adherence capacity to healthcare system delivery capability. This shift from post-initiation behavioral barriers to pre-initiation structural barriers necessitates corresponding innovations in cascade conceptualization, measurement frameworks, and intervention design.

Equity implications are profound. Populations facing the greatest structural barriers—PWID (projected 20–30% bridge period completion), adolescents (30–40%), and women (40–50%)—experience the lowest bridge period completion rates, threatening to widen HIV prevention disparities despite LAI-PrEP’s superior efficacy. Paradoxically, these populations demonstrate the greatest potential benefit from targeted interventions during the bridge period. Computational modeling demonstrates that evidence-based bridge period management could improve global initiation success from approximately 24% to 44%, with PWID showing 265% relative improvement and adolescents showing 147% improvement [13]. Addressing bridge-period barriers is thus both an equity imperative and an implementation efficiency opportunity.

Evidence-based strategies operate through complementary mechanisms: eliminating bridge periods through oral-to-injectable transitions, compressing them through accelerated testing, navigating them through structured support, and removing structural barriers through integrated service delivery. Conservative initiation protocols serve important safety functions given LAI-PrEP’s pharmacokinetic irreversibility. The implementation challenge is not abandoning safety but streamlining processes sufficiently to retain motivated individuals during necessary waiting periods.

Realizing LAI-PrEP’s potential requires systematic attention to prescription-to-protection gaps. The traditional cascade must be reconceptualized to explicitly recognize bridge period navigation as a distinct, measurable implementation step. Ongoing implementation trials (HPTN 102, HPTN 103) will generate population-tailored intervention evidence. Companion computational validation [13] demonstrates how clinical decision support tools can operationalize bridge period management strategies across diverse clinical contexts and populations, providing a scalable pathway for systematic implementation. By addressing this structural gap through infrastructure investment, protocol innovation, and equity-focused approaches, LAI-PrEP can be transformed from a clinically proven but underutilized intervention into a cornerstone of HIV prevention, realizing its promise for all populations who need it.

## Figures and Tables

**Table 1:** Formal Definition of the LAI-PrEP Bridge Period.

Component	Description
**Definition**	The temporal interval and associated procedural requirements between prescription (start point) and first injection administration (end point).
**Duration determinants**	HIV testing strategy employed (antigen/antibody vs. dual antigen/antibody + RNA); time elapsed since last potential HIV exposure; optional oral lead-in period; insurance prior authorization timelines; appointment scheduling availability.
**Clinical significance**	During this interval, individuals maintain prevention motivation but lack pharmacological protection, creating a structural vulnerability unique to LAI-PrEP that is absent in oral PrEP formulations.

**Table 2:** Population-specific bridge period evidence summary. Projected completion rates are derived from the 47% baseline attrition (CAN Community Health [11]) with documented barrier amplifiers. Computational validation at UNAIDS target scale (21.2 million individuals) confirmed these estimates [13].

Population	Key Barriers	Projected Completion	Supporting Evidence
Adolescents (16–24)	Temporal discounting; privacy/EOB concerns; limited autonomous navigation	30–40%	CDC surveillance [19]; delay discounting [20,21]; medical mistrust [22]; adolescent PrEP barriers [17,18,23,24,25]; PURPOSE-1 adolescent data [5]
Women	Transportation [26]; childcare [27]; medical mistrust (39%); intimate partner violence [28,29,30]; oral PrEP barriers	40–50%	PrEP in justice-involved women [31]; LA-ARV perceptions [32]; IPV and PrEP [28,29,30]; WWID product choice [33]; oral PrEP barriers [23,34,35]; trial efficacy [2,4,5]
PWID	Criminalization (17% avoiding care) [36]; housing instability [37]; low awareness (2% using PrEP) [38,39,40]; healthcare stigma	20–30%	UNODC report [41]; WHO PWID guidelines [42]; incarceration-HIV nexus [36]; stigma [43,44,45]; county vulnerability [46]; housing [37]; syringe services [47]; trial equity [48]; PrEP cascade [39]
Transgender & gender-diverse	Healthcare discrimination; economic marginalization; hormone interaction concerns	35–45%	WHO trans health [49]; gender-affirming care [50,51]; PrEP adherence [52]; trial data [3,6]
MSM	47% baseline attrition despite highest uptake; insurance/scheduling barriers	53% (baseline)	CAN Community Health [11]; Trio Health persistence [12]
Global (Sub-Saharan Africa)	Cold chain; workforce capacity; task shifting needs; 62% of global PrEP need	21.7% (modeled baseline)	UNAIDS 2024 [53]; WHO task shifting [54]; computational validation [13]

**Table 3:** Evidence-based intervention library for LAI-PrEP bridge-period navigation (n = 21). Interventions are classified by mechanism (eliminate, compress, navigate, remove barriers, clinical support, system-level) with evidence tier ratings (Tier 1: RCTs/large cohorts; Tier 2: observational/implementation studies; Tier 3: emerging/conference data). Effect sizes represent the estimated absolute improvement in bridge period completion rate.

Intervention	Mechanism	Effect Size	Evidence Source	Tier	Complexity
**ELIMINATE THE BRIDGE PERIOD**					
Oral-to-injectable same-day switching	Eliminate bridge	+35% abs.	CAN Community Health [11]; Trio Health [12]	1	Low
**COMPRESS THE BRIDGE PERIOD**					
HIV-1 RNA testing	Compress bridge	+15–20%	WHO 2025 [57]; CDC [58]	2	Medium
Rapid lab turnaround (24–48 h)	Compress bridge	+10–15%	CDC guidelines [58]	2	Medium
Point-of-care HIV testing	Compress bridge	+8–12%	FDA-approved platforms	3	High
**NAVIGATE THE BRIDGE PERIOD**					
Dedicated patient navigation	Navigate bridge	+12–20%	SF PrEP navigation [59,60,61]; cancer care [26]	1	Medium
Peer navigation	Navigate bridge	+15–20%	HIV cascade studies [62]; PrEP uptake [63]	2	Medium
SMS/text reminders	Navigate bridge	+10–15%	Healthcare meta-analyses [64]	1	Low
Population-tailored navigation	Navigate bridge	+20–30%	PWID literature [18,65]; HPTN 083-02 [66]	2	Medium–High
**REMOVE FINANCIAL & LOGISTICAL BARRIERS**					
Transportation support	Remove barriers	+10–15%	Cancer care [26]; PrEP barriers [67]	2	Low–Medium
Childcare assistance	Remove barriers	+8–12%	Family planning parallels [68]	3	Medium
Mobile delivery services	Remove barriers	+15–25%	Community delivery [69]; WHO 2025 [57]	2	High
Bundled payment models	Structural support	+12–18%	Episode-based payment theory [67]	3	High
Accelerated insurance authorization	Structural support	+12–15%	Prior authorization policy [62,70]	3	Medium
**ADDRESS CLINICAL & INTERPERSONAL BARRIERS**					
Medical mistrust intervention	Clinical support	+8–12%	CHW navigation [34,71]	2	Medium
Anti-discrimination protocols	Clinical support	+10–15%	SGM healthcare [38]	2	Low–Medium
Confidentiality protections	Clinical support	+8–12%	Adolescent PrEP [35]	2	Medium
Language-concordant services	Clinical support	+10–12%	Language access studies [72]	2	Medium
**SYSTEM-LEVEL REDESIGN**					
Telemedicine integration	System-level	+10–15%	Telehealth expansion [73]	2	Medium
Pharmacist-led prescribing	System-level	+15–20%	Pharmacist PrEP studies [74]	2	High
Harm reduction integration (PWID)	System-level	+25–35%	SSP-integrated services [38,55]	2	Medium–High
Community-based delivery	System-level	+15–25%	Community models [75]; WHO 2025 [57]	2	High

## Data Availability

Data and code supporting this manuscript are available at: GitHub: https://github.com/Nyx-Dynamics/lai-prep-bridge-tool-pub, (accessed on 1 March 2026); Zenodo: https://zenodo.org/record/17873201, (accessed on 1 March 2026).

## Supplementary Materials

The following supporting information can be downloaded at: https://www.mdpi.com/article/10.3390/v18030336/s1. File S1: CLINICAL TRIAL EVIDENCE; File S2: COMPLETE INTERVENTION INVENTORY; File S3: GLOBAL IMPLEMENTATION; File S4 IMPLEMENTATION STRATEGIES; File S5: POPULATION GUIDANCE; File S6: RESEARCH AGENDA.
